# Distending Pressure Did Not Activate Acute Phase or Inflammatory Responses in the Airways and Lungs of Fetal, Preterm Lambs

**DOI:** 10.1371/journal.pone.0159754

**Published:** 2016-07-27

**Authors:** Rebecca Y. Petersen, Emily Royse, Matthew W. Kemp, Yuichiro Miura, Andres Noe, Alan H. Jobe, Noah H. Hillman

**Affiliations:** 1 Division of Neonatology, Cardinal Glennon Children’s Hospital, Saint Louis University, Saint Louis, MO, 63104, United States of America; 2 Division of Pulmonary Biology, Cincinnati Children’s Hospital Medical Center, University of Cincinnati, Cincinnati, OH, 45229, United States of America; 3 School of Women and Infants’ Health, University of Western Australia, Perth, WA, 6009, Australia; University of Giessen Lung Center, GERMANY

## Abstract

**Background:**

Mechanical ventilation at birth causes airway injury and lung inflammation in preterm sheep. Continuous positive airway pressure (CPAP) is being increasingly used clinically to transition preterm infants at birth.

**Objective:**

To test if distending pressures will activate acute phase reactants and inflammatory changes in the airways of fetal, preterm lambs.

**Methods:**

The head and chest of fetal lambs at 128±1 day GA were surgically exteriorized. With placental circulation intact, fetal lambs were then randomized to one of five 15 minute interventions: PEEP of 0, 4, 8, 12, or 16 cmH_2_O. Recruitment volumes were recorded. Fetal lambs remained on placental support for 30 min after the intervention. The twins of each 0 cmH_2_O animal served as controls. Fetal lung fluid (FLF), bronchoalveolar lavage fluid (BAL), right mainstem bronchi and peripheral lung tissue were evaluated for inflammation.

**Results:**

Recruitment volume increased from 0.4±0.04 mL/kg at 4 cmH_2_O to 2.4±0.3 mL/kg at 16 cmH_2_O. The lambs were surfactant deficient, and all pressures were below the opening inflection pressure on pressure-volume curve. mRNA expression of early response genes and pro-inflammatory cytokines did not increase in airway tissue or lung tissue at any pressure compared to controls. FLF and BAL also did not have increases in early response proteins. No histologic changes or Egr-1 activation was present at the pressures used.

**Conclusion:**

Distending pressures as high as 16 cmH_2_O did not recruit lung volume at birth and did not increase markers of injury in the lung or airways in non-breathing preterm fetal sheep.

## Introduction

The transition at birth from fluid filled airspaces to aeration of the lungs is fundamental to mammalian life [1]. This transition requires a coordinated clearance of fetal lung fluid, surfactant secretion and development of consistent breathing patterns to be successful [2]. The initial clearance of lung fluid from the airways requires the generation of large initial negative pressure breaths [1, 3]. Surfactant deficient preterm animals and humans may lack the muscle strength to generate sufficient negative pressures to overcome their compliant chest wall and high airway surface tension [2]. Consequently, many preterm infants require assistance with the transition from fetal life [4, 5]. The preterm lung can be easily injured by the assistance required, and the resultant lung inflammation may contribute to the progression towards bronchopulmonary dysplasia (BPD) [6, 7]. Mechanical can injure the preterm airways and contributes to the airway hyperactivity seen in infants with a history of BPD [8–10]. Despite the increasing survival of preterm infants, there have been no major changes in survival without morbidity over many years [4, 11]. New strategies to aid infants in the birth transition are needed.

Airway epithelial injury can begin with the initiation of ventilation at birth and is likely due to the rapid movement of lung fluid-air interface in the airways. The injury cascade takes only a few breaths to begin, as even a very brief large tidal volume ventilation can initiate an injury that is then perpetuated by continued ventilator support [12, 13]. The unequal distribution of tidal volumes within the lung and the repetitive opening and closing of airspaces also contribute to the lung injury from mechanical ventilation of the lungs of the preterm [10, 14, 15]. Stretching the preterm airways also leads to release of acute phase proteins (HSP70) that can activate inflammation [8, 16]. Efforts to recruit functional residual capacity and decrease fluid in airways with a prolonged sustained inspiration (SI) are being evaluated in preterm animals and infants. Unfortunately SI did not protect the lungs of preterm sheep from injury from mechanical ventilation; in fact SI alone caused a modest injury in surfactant deficient lambs [17–20]. The use of positive end-expiratory pressure (PEEP) to maintain alveolar patency is beneficial in the neonatal intensive care unit [21]. In preterm lambs, ventilation without PEEP causes airway injury and lung inflammation that can be in part mitigated with the addition of PEEP [22]. Recruitment of the lung with gradually increasing PEEP during ventilation also decreased lung injury in preterm sheep [23, 24]. In preterm, surfactant-treated lambs, a PEEP of 4 cmH_2_O was protective against lung injury compared to 0 cmH_2_O, but lungs of animals on PEEP 7 cmH_2_O had neutrophil and protein injury responses similar to no PEEP [17, 25]. Ventilatory support via an endotracheal tube (invasive support) is injurious to the lungs of surfactant deficient preterm animals, but it is unclear if non-invasive support also follows this trend.

Several studies support the recommendation to use non-invasive strategies following birth to assist in the transition from fetal life [26, 27]. Even with the use of early CPAP in the delivery room, the decrease in the incidence of BPD is modest at about 10% [28, 29]. In clinical care non-invasive strategies are being used with more frequency to transition infants at birth and higher CPAP pressures are used to try to avoid intubation. Therefore, it is important to evaluate if higher airway pressures (PEEP) can injure the preterm airways or lung. In one report, preterm lambs given PEEP alone had a modest increase in inflammatory markers, but the observation was not systematically explored [17]. The lack of a protective effect of PEEP 7 cmH_2_O compared to 4 cmH_2_O in ventilated newborn lambs also raises some concern [25]. Using a preterm fetal lamb model, which avoids ventilation and oxygen exposure, we tested the hypothesis that higher PEEP pressures cause airway injury and lung inflammation in non-breathing sheep.

## Methods

### Fetal preparation for PEEP intervention

This study was approved by the Animal Ethics Committees of the University of Western Australia, Saint Louis University, and Cincinnati Children's Hospital Medical Center.

Date mated Merino Ewes at 128 ±1 days gestational age (GA; term is about 150 days GA) were premedicated with 0.5 mg/kg of IM xylazine, 5 mg/kg of IV ketamine, and 0.25 mg/kg IV midazolam. The ewes were intubated and maintained during the procedure with anesthesia with isofluorane (0.5–2% in 100% O_2_). The isofluorane crosses the placenta, anesthetizes the fetus, and eliminates fetal breathing [17]. The fetal head and chest were exteriorized through a midline hysterotomy, with maintenance of placental blood flow [12]. The fetal lamb then received a tracheostomy to secure a 4 F endotracheal tube. Free-flowing fetal lung fluid (FLF) was gently removed with a 7 F catheter (average 13 ml/kg fetal weight) and an aliquot was snap frozen. The endotracheal tube was attached to a Neopuff (Fisher & Paykel, New Zealand) with a PEEP valve to adjust pressure. In line with the endotracheal tube, we placed a 1.3 mL pneumotach (RSS HR100 system, Hans Rudolph, Kansas City, MO) to continuously record the volume recruitment over time. Flow sensor was calibrated daily with manufacturer’s syringe and previously correlated well with respiratory inductance plethysmography [17].

### Fetal Interventions

The fetal lambs were randomly assigned to one of five groups for a 15 minute intervention: 1) a PEEP of 0 cmH_2_O; 2) a PEEP of 4 cmH_2_O; 3) a PEEP of 8 cmH_2_O; 4) a PEEP of 12 cmH_2_O; or 5) a PEEP of 16 cmH_2_O with heated, humidified 100% N_2_ to avoid oxygen exposure. After the 15 minute intervention, the endotracheal tube was occluded and the fetus was returned to the uterus and maintained on placental circulation for an additional 30 minutes. The fetus was then euthanized with 100 mg/kg of pentobarbital, and fetal lung fluid (FLF) was collected and snap frozen. Cord blood gasses were measured using a Siemens Rapidlab 1265 (Siemens, Australia). The 45 minute experimental period was chosen to optimize the detection of mRNA for acute phase inflammatory response genes [30]. The twin lambs of the 0 cmH_2_O animals received maternal anesthesia but no intervention and were used as additional controls.

### Lung volumetrics, processing and BAL analysis

Inflation and deflation pressure-volume curves were measured with a stepwise change in pressure to a maximum of 40 cmH_2_O [31]. Bronchioalveolar lavage fluid (BALF) of the left lung was collected by repetitive saline lavage. FLF and BALF were used for measurement of total protein [32] and sandwich ELISA assays for Heat shock protein (HSP) 70 and HSP60 in the airway fluids (R&D Systems, USA) [22]. Tissues from the peripheral regions of the right lower lobe near the pleural edge and the right mainstem bronchus at approximately 3^rd^ branch point were snap frozen for RNA and protein isolation [33].

### Histologic evaluation

Immunohistochemistry protocols used 5 μm paraffin sections of formalin inflation fixed lung tissues that were pre-treated with 3% hydrogen peroxide to inactivate endogenous peroxidases [34, 35]. The sections were incubated overnight with anti-human Egr-1 with 1:250 dilution (Santa Cruz, USA), in 4% normal goat serum, followed by biotin labeled secondary antibody. Immunostaining was visualized by Vectastain ABC Peroxidase Elite kit (Vector Laboratories Inc, USA). The antigen detection was enhanced with nickel-DAB, followed by TRIS-cobalt and the nuclei counterstained with nuclear fast red [34]. Hematoxylin and Eosin stained tissues were blinded and evaluated for airway injury and inflammation, as previously described [17].

### Quantitative RT-PCR

Messenger RNA was extracted from lung tissue from the right mainstem bronchus and peripheral lung tissue with TRIzol (Invitrogen, Grand Island, NY). cDNA was generated from 1 mg mRNA using Verso cDNA kit (Thermoscientific, USA). We used custom Taqman gene primers (Life technologies, USA) for ovine sequences for Interleukin 1β (IL-1β), IL-6, MCP1, early growth response protein 1 (Egr-1), and connective tissue growth factor (CTGF) [22, 36]. Quantitative Real Time PCR was performed using iTaq Universal mix (BioRad, USA) in a 15 μl reaction on a CFX Connect machine and software (BioRad, USA). 18S primers (Life Technologies, USA) were used for the internal loading control. Results are reported as fold increase over mean for all of the intervention groups.

### Western blot analysis

Protein concentrations from lung tissue were determined using Bio-Rad Protein Assays [37]. 40 μg of protein was denatured in BME at 95 degrees for 5 minutes, then run on Tris-glycine 10% gel and transferred to 0.45 μm nitrocellulose membrane (Bio-rad, USA). Membranes were blocked in 5% normal milk fat and then incubated with Egr-1 1:500 (Santa Cruz, USA) or β−Actin 1:2000 (Thermoscientific, USA) overnight at 4 degrees. Appropriate IgG-HRP secondary antibodies were applied at 1:10000 dilutions. Membranes were developed with ECL (Pierce/ThermoFisher) and then imaged on Syngene PXi multi-gel imaging system (Syngene, USA) and quantified with ImageJ 1.48V (N.I.H., USA). Egr-1 and β−Actin were analyzed on the same gels without membrane stripping.

### Data analysis and statistics

Results are shown as mean (StDev) and reported as fold increase over the mean. Statistics were analyzed using Prism 6 (GraphPad, USA) using Student’s *t*-test, Mann-Whitney non-parametric, or ANOVA tests as appropriate. Significance was accepted as p<0.05.

## Results

**Fig 1:**
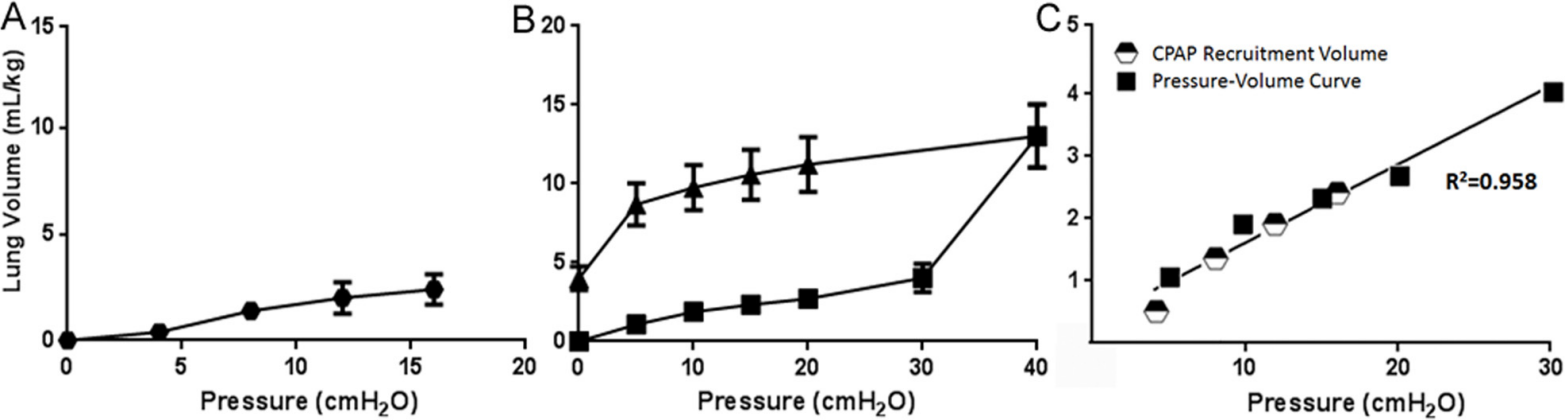
Lung Volume Recruitment and post-mortem pressure volume cures. (A) Average lung volume recruited at the end of intervention period plotted against PEEP pressure demonstrates a slight increase in ml/kg with increased pressures. (B) Post-mortem mean pressure-volume curves for study animals with chests open. (C) Pressure-volume relationship during recruitment maneuver (half circles) and from post-mortem pressure-volume curve (squares) show strong correlation demonstrating little effect of the closed chest wall and all PEEP pressures below the pressure at which the unrestricted lung expands.

All fetal lambs survived the intervention and 30 minute recovery period on placental support. The gestational age (128±0.3 days), birth weight, gender and cord gases were similar between groups, with the exception of the 16 cmH_2_O group which was slightly heavier (Table 1). There were no differences in lung weights per kg (Table 1), suggesting no increase in lung edema or clearance with pressure exposure. In anesthetized fetal sheep without spontaneous breathing, the PEEP pressures recruited very small lung volumes over 15 min (less than 3 ml/kg), even at a pressure of 16 cmH_2_O (Fig 1A). All of the lambs were very surfactant deficient (Table 1). The post mortem lung gas volumes at 40 cmH_2_O were not different between the PEEP groups. The inflection point for the post-mortem lungs to inflate was over 30 cmH_2_O (Fig 1B). The correlation between pressures and volumes recruited with 15 minutes of PEEP and the inflation arm of the pressure-volume curve with an open chest were high (R^2^ = .96) (Fig 1C), demonstrating that chest wall constriction during PEEP did not limit lung expansion. There were no differences between surgical controls and 0 cmH_2_O animals, and values for markers of injury are reported as fold increase over the combined control groups.

**Table 1:** Animal characteristics, protein secretion, and surfactant levels.

Group	n	Birth Weight (kg)	M:F	Cord pH	FLF Protein (mg/ml)	Surfactant (μmol/kg)	Left Lung Weight (g/kg)
**Controls**	7	3.1 ± 0.5	2:5	7.29 ± 0.09	0.15 ± 0.03	1.09 ± 1.3	11.9 ± 1.1
**0 cmH**_**2**_**O**	6	3.1 ± 0.4	3:3	7.30 ± 0.03	0.25 ± 0.10	0.91 ± 0.94	11.8 ± 0.4
**4 cmH**_**2**_**O**	9	3.1 ± 0.4	5:4	7.29 ± 0.07	0.17 ± 0.08	1.00 ± 0.69	13.1 ± 0.5
**8 cmH**_**2**_**O**	8	3.2 ± 0.4	3:5	7.29 ± 0.03	0.16 ± 0.08	0.75 ± 0.67	12.2 ± 0.6
**12 cmH**_**2**_**O**	8	3.2 ± 0.3	5:3	7.28 ± 0.10	0.22 ± 0.09	1.34 ± 1.30	13.6 ± 0.6
**16 cmH**_**2**_**O**	8	3.8 ± 0.5*	4:4	7.27 ± 0.04	0.18 ± 0.07	0.26 ± 0.13	12.6 ± 0.5

Mean±SD

*p<0.05 vs controls

### Fetal lung fluid and bronchioalveolar lavage fluid

Increasing distending pressures did not increase total protein secretion in the fetal lung fluid collected at the end of the recovery period or the BALF compared to control animals (Table 1). The BALF also did not have increased surfactant levels following intervention. There were no differences between groups for release of acute phase proteins (HSP 60, HSP 70) in the FLF after the intervention or BALF (data not shown).

### Cytokine and acute phase responses in peripheral lung and right mainstem bronchus

The intervention groups did not have significant differences in mRNA for IL-1β, IL-6 or MCP1 in the peripheral lung tissue or the right mainstem bronchus relative to controls (Table 2). There were also no significant differences in the acute phase response genes, Egr-1 or CTFG in either of these tissues. There were no differences on blinded evaluation of Hematoxylin and Eosin staining of lung sections. Relative to controls, no increased expression of Egr-1 was apparent on immunohistochemistry staining of airways (data not shown). Furthermore, Western Blot did not show increased expression of Egr-1 protein (data not shown).

**Table 2:** Pro-inflammatory cytokine and acute phase gene mRNA expression.

	Right Mainstem Bronchus				Peripheral Lung Tissue			
Group	IL-1β	IL-6	Egr-1	CTGF	IL-1β	IL-6	Egr-1	CTGF
**Controls**	1.0±0.2	1.0±0.2	1.0±0.3	1.0±0.2	1.0±0.2	1.0±0.2	1.0±0.3	1.0±0.2
**4 cmH**_**2**_**O**	0.8±0.1	0.9±0.1	0.8±0.1	0.7±0.1	1.2±0.2	1.7±0.4	1.0±0.2	1.4±0.2
**8 cmH**_**2**_**O**	1.1±0.2	1.1±0.2	1.1±2.0	1.0±0.2	1.3±0.4	1.7±0.4	1.0±0.2	1.4±0.4
**12 cmH**_**2**_**O**	0.9±0.3	1.0±0.3	0.9±0.2	1.1±0.4	1.0±0.2	1.3±0.3	1.0±0.2	1.3±0.3
**16 cmH**_**2**_**O**	1.4±0.4	1.4±0.3	1.1±0.4	1.9±1.0	1.0±0.2	1.8±0.4	1.0±0.2	1.4±0.3

## Discussion

We found no effect of PEEP pressures from 0 to 16 cmH_2_O on markers of lung injury with a preterm fetal model of surfactant deficient lambs. These measurements should be sensitive indicators because the controls have no baseline inflammation and a maneuver such as a prolonged sustained inflation can elicit easily detected inflammation in this model [17]. Although contrary to our hypothesis, these results should be reassuring to clinicians choosing to use higher levels of CPAP to avoid intubation of preterm infants. In these poorly compliant lamb lungs, due to moderate prematurity and no antenatal steroid exposure, fifteen minutes of distending pressures up to 16 cmH_2_O did not recruit gas effectively to the lungs. There was a dose response curve noted as more pressure inflated more of the lung tissue, but at the highest pressure used, 16 cm H_2_O, only ~3 ml/kg of lung gas volume was achieved. As measured by the lung volume curves at necropsy (Fig 1B and 1C), this pressure did not effectively open the lungs. Although the animals in the 16 cmH_2_O group were slightly heavier, the lung to body weight ratio was similar and pressure should have been dispersed throughout lung thus not affecting markers of injury. The air that entered the lungs likely did not go beyond the larger airways, as we removed 13 ml/kg of fetal fluid prior to the application of PEEP. We thought that this airway inflation has the potential to initiate an inflammatory cascade because ventilation injury to the preterm lung is initiated in the airways [8]. Furthermore, HSP70, a marker of airway injury,was secreted from newborn lamb trachea when exposed to mechanical ventilation [16]. Also mechanical ventilation changes the tissue matrix with disruption of epithelial integrity in the lamb trachea [38]. It would follow that stretching of the lamb airways in this model could have initiated inflammation and airways injury. Contrary to this hypothesis, our results demonstrate no differences between airways containing 3 ml/kg of volume and 0 ml/kg of volume.

The lack of injury response in these lambs can be further put in context of the effects of mechanical ventilation or sustained inflation alone. Previously a 20 second SI at 50 cmH_2_O recruited a lung volume around 15 ml/kg in surfactant deficient fetal lambs, and this single large inflation alone initiated the process of lung inflammation and injury [17]. Other groups have found a SI to 7 ml/kg increased Egr-1 mRNA [39]. Fetal lambs treated with surfactant and smaller recruitment volumes (7 ml/kg) did not demonstrate the negative effects of SI [18, 39]. In previous studies of mechanical ventilation in the preterm lamb model, the normal lamb tidal volume of 7 ml/kg generated lung inflammation and Egr-1 activation surrounding the small airways and terminal bronchioles [36]. Mechanical ventilation with moderate tidal volumes also causes the release of heat shock proteins into the airway, which can worsen the inflammatory response through activation of toll-like receptors [36]. The higher levels of pro-inflammatory cytokines and Egr-1 activation seen in previous animals on PEEP 8 cmH_2_O, a PEEP pressure used in clinical studies, was not substantiated in this larger study [17, 40]. The largest difference between these studies was the amount of fetal lung fluid removed prior to intervention, with 13 ml/kg removed in current study compared to 8 ml/kg in study demonstrating injury effect [17]. The additional lung fluid in previous lambs, as evident by a higher left lung weight of 15 g/kg, may have increased the injury seen due to sheering effects on epithelium [8, 17]. Similar fetal models have removed 15 ml/kg of fetal fluid and used 8 cmH2O without an effect on inflammatory markers [23, 24]. The largest lung volumes recruited in this study did not even reach half of the moderate tidal volume previously shown to be injurious or the functional residual capacity recruited by a brief sustained inflation [17, 18, 36]. Although not tested, higher PEEP values above the lower inflection point of the lung expansion, 30 cmH_2_O, would likely have opened the lung and caused similar effects to a single sustained inflation. These sheep were not treated with antenatal steroids, thus there may have been different effects in a more compliant lung without as much lung fluid [41, 42]. Since the fetal animals are not breathing, spontaneously breathing in humans with higher PEEP could still cause injury.

Our results support the concept that lung injury is more likely caused by increased volume rather than barotrauma from high pressures. Several studies have addressed this fundamental question. In a rabbit model of high pressure lung injury, rabbits had chest casts placed to limit volume expansion of the chest and were exposed to high ventilator inspiratory pressures [43]. The casted animals, when compared to animals who did not have restriction to lung volume recruitment, did not have significant increases in signs of microvascular damage [43]. Similar studies in young lambs with their chest and abdomen bound to limit chest wall excursion during mechanical ventilation had decreased protein secretion and altered lymphatic changes compared to lambs with unrestricted tidal volumes [44]. Rats with abdominal and chest banding had similar lung protection when lung volume was limited [45]. These studies support the argument that increased volume causes the injury and inflammatory cascade in developing lungs, and not the effect of an absolute pressure. The distending pressures used in our current study did not result in significant lung expansion and thus the injury and the inflammatory cascade was not initiated.

We conclude that increasing distending pressure alone, when it does not result in significant lung expansion, does not trigger the injury response in the airways and lung. Further evaluation of the effects of CPAP or distending pressures in a breathing animal model would further strengthen these conclusions.

## Supporting Information

S1 Dataset 2014 Resuscitation data.xlsl contains primary data from study. (XLSX)
